# Complex Variant of Philadelphia Translocation Involving Chromosomes 9, 12, and 22 in a Case with Chronic Myeloid Leukaemia

**DOI:** 10.1155/2014/691630

**Published:** 2014-06-18

**Authors:** F. Malvestiti, C. Agrati, S. Chinetti, A. Di Meco, S. Cirrincione, M. Oggionni, B. Grimi, F. Maggi, G. Simoni, F. R. Grati

**Affiliations:** ^1^Research and Development, Cytogenetics and Molecular Biology, TOMA Advanced Biomedical Assays S.p.A., 25/27 Francesco Ferrer Street, 21052 Busto Arsizio, Varese, Italy; ^2^Treviglio Caravaggio Hospital, 1 Pittori Cavenaghi Square, 24043 Caravaggio, Bergamo, Italy

## Abstract

Chronic myeloid leukemia (CML) is a hematopoietic stem cell disorder included in the broader diagnostic category of myeloproliferative neoplasms, associated with fusion by BCR gene at chromosome 22q11 to ABL1 gene at chromosome 9q34 with the formation of the Philadelphia (Ph) chromosome. In 2–10% of CML cases, the fusion gene arises in connection with a variant translocation, involving chromosomes 9, 22, and one or more different chromosomes; consequently, the Ph chromosome could be masked within a complex chromosome rearrangement. In cases with variant Ph translocation a deletion on der(9) may be more frequently observed than in cases with the classical one. Herein we describe a novel case of CML with complex variant Ph translocation involving chromosomes 9, 12, and 22. We present the hematologic response and cytogenetic response after Imatinib treatment. We also speculated the mechanism which had originated the chromosome rearrangement.

## 1. Introduction

Chronic myeloid leukemia (CML) is a hematopoietic stem cell disease included in the broader diagnostic category of myeloproliferative neoplasms [1] that is characterized by neoplastic overproduction of mainly granulocytes. CML is consistently associated with fusion by chromosome translocation of the breakpoint cluster region gene (*BCR*) at chromosome 22q11 to the Abelson gene (*ABL1*) at chromosome 9q34. This fusion gene BCR/ABL1 encodes for an oncoprotein (P210, more rarely P190 or P230) with a strong constitutive activated tyrosine kinase activity inducing several downstream signals causing the transformation of hemopoietic stem cells [2]. The translocation t(9;22) may be detected by routine karyotype as Philadelphia (Ph) chromosome, although in 2–10% of the cases, the fusion gene arises from a variant translocation [3]. Two variant subgroups have been recognized: the simple variant group with the 22q segment translocated on chromosome other than 9 and the complex variant translocation involving chromosomes 9, 22, and one or more additional chromosome/s. Consequently, the Ph chromosome could be masked within a complex chromosome rearrangement. Although all chromosomes could be involved in these variant translocations, there is a marked clustering to specific chromosomal bands suggesting that specific regions are particularly prone to breakage. In addition, in variant cases a deletion on der(9) may be more frequent than in cases with the classical Ph translocation (40% versus 14%) [4]. Prognostic evaluation of different complex variants was attempted in a limited number of CML cases giving controversial and inconclusive results [5]. Herein we describe a novel CML case with complex variant Ph translocation involving chromosomes 9, 12, and 22. We evaluated the response to the Imatinib treatment and speculated the molecular events underlying this chromosome rearrangement.

## 2. Case Report

**Figure 1:**
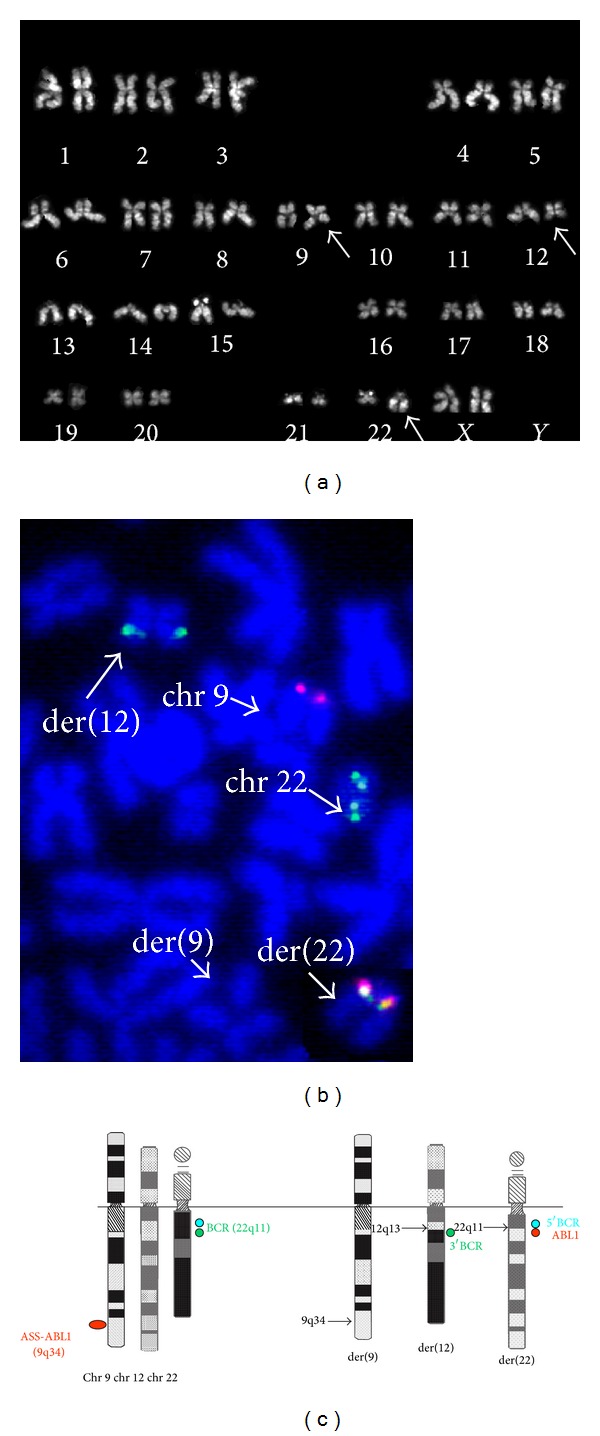
(a) QFQ karyotype derived from bone marrow cells. The arrows indicate the derivative chromosomes involved in the rearrangement. (b) BCR/ABL1 FISH signal pattern on metaphase. The arrows indicate the rearranged chromosomes and the normal chromosomes 9 and 22. (c) Ideogram of the rearrangement identified in our CML case with the schematic representation of the FISH probe signals.

The patient, a 72-year-old woman, had a clinical history of immune-mediated thrombocytopenia. During routine laboratory analysis, an unexpected increase of white blood count (WBC) was found and a CML was suspected. The laboratory data showed a WBC count of 39.2 × 10^3^/mcL, with 60% of neutrophils, 21% of lymphocytes, 10% of monocytes, 2% of eosinophils, 2% of basophils, 4% of myelocytes, and 1% of metamyelocytes. Hemoglobin concentration of 13.5 g/dL was within the normal range, while the platelet count was low (101 × 10^3^/mcL). Cytogenetic analysis on bone marrow and RT-PCR on peripheral blood were carried out. Conventional cytogenetic analysis was performed on unstimulated 24- and 48-hour bone marrow cultures. Cells were cultured and processed by standard methods [6] and chromosomes were stained by QFQ-banding. The analysis was performed according to the Italian and European Acquired Cytogenetics and the ESMO (European Society of Medical Oncology) clinical practice guidelines [7–9]. FISH analysis using BCR/ABL1 t(9;22) Triple-Color and Dual-Fusion probe and Sub-Telomere 9qter probe (Kreatech Diagnostics Vlierweg 20, 1032 LG Amsterdam, The Netherlands) was done following the manufacturer procedures. Karyotype result was described according to the ISCN 2013 [10]. Reverse-transcription quantitative polymerase chain reaction (RT-PCR) for chimeric BCR-ABL1 transcript on peripheral blood was performed with Philadelphia p210 Q-PCR Alert kit (Nanogen Inc., San Diego, CA, USA), based on TaqMan technology. RNA extraction and RT-PCR were performed following the insert kit instructions (Nanogen Inc., San Diego, CA, USA). The measurement of the cDNA of P210 was normalized to the cDNA of ABL1 gene. Conventional cytogenetic analysis on bone marrow showed on 22 metaphases a reciprocal translocation involving the long arm of chromosomes 12 and 22, t(12;22), without the involvement of chromosome 9 (Figure 1(a)). The presence of a cryptic BCR/ABL1 fusion transcript was detected by RT-PCR and subsequently by interphase FISH analyses on bone marrow. Quantitative RT-PCR analysis for BCR/ABL1 on peripheral blood revealed the major chimeric transcript, with a BCR-ABL1(P210)/ABL1 ratio of 14.95% (International Scale). FISH analysis with BCR/ABL1 t(9;22) Triple-Color and Dual-Fusion probe was performed to characterize the t(12;22) translocation and to detect the localization of the fusion gene. The probe set is a mixture of ASS-ABL1 probe labeled in red and of BCR probe with the proximal BCR region labeled in blue and the distal one in green. FISH on 200 metaphases and nuclei showed the following: (i) one purple (blue/red) fusion signal representing the fusion gene (BCR/ABL1) on der(22), (ii) one green signal of 3′ BCR sequences on chromosome 12 involved in translocation t(12;22), (iii) a green/blue signal on normal chromosome 22, and (iv) a red signal on normal chromosome 9 (Figures 1(b) and 1(c)). The reciprocal fusion ABL1/BCR signal was not detected. FISH analysis on 200 nuclei and metaphases using the subtelomeric 9qter probe was performed to further investigate the involvement of chromosome 9 in the complex rearrangement: it showed a normal signal pattern. In summary, FISH disclosed the deletion of the 5′ ABL1 sequences, including the ASS gene, on der(9), and allowed to map the breakpoint of t(12;22) within the sequences distal to BCR gene. The BCR probe gave a splitted signal on der(22) and on der(12), respectively. The ISCN karyotype was 46,XX,der(9)del(9)(q34q34)ins(22;9)(q11.2;q34q34),der(12)t(12;22)(q13;q11.2),der(22)ins(22;9)t(12;22)[22]. All these results were consistent with the CML diagnosis and the patient started the treatment with Imatinib mesylate (Glivec). After three months of therapy, the WBC count was 5.1 × 10^3^/mcL, with 49.7% of neutrophils, 37.8% of lymphocytes, 7.6% of monocytes, 4.3% of eosinophils, 0.6% of basophils, the hemoglobin concentration was 12.4 g/dL, and platelets count was 211 × 10^3^/mcL. The molecular cytogenetic followup by interphase FISH with BCR/ABL1 probe on 200 nuclei, after 4 and 6 months of therapy, showed a normal signal pattern, while the chromosome analysis at six months revealed a new abnormal clone detected in the 5% (2 out of 5 metaphases and 10 out of 200 interphase nuclei analyzed by FISH with chromosomes 8 and 9 centromeric probes) of the sample with trisomies 8 and 9 (48,XX,+8,+9).

## 3. Discussion

We describe a patient with CML associated with a novel cryptic complex variant t(9;22), involving chromosome 12 besides chromosomes 9 and 22, which was unmasked and characterized by RT-PCR and FISH analyses. In agreement with ESMO clinical practice guidelines, this case report proves the role of these molecular approaches in detecting cryptic fusion gene in some types of variant translocations with masked Ph and der(9) chromosomes. As previously reported, the breakpoints location of complex variant t(9;22) is nonrandom with a marked clustering to specific chromosome bands suggesting that some regions are more prone to breakage. This finding could be explained by the presence of a specific genomic structure mediating the recombination. Indeed a significant clustering was described for high CG content regions, Alu repeats, LINE, genes, and miRNA explaining the presence of recombination hotspots [11, 12]. The 12q13 chromosome region, involved in our case, was described by Costa et al. [13] in association with complex Philadelphia translocation and in some cases of three-way translocation t(9;22) [11]. In addition, this region is involved both in other chromosomal translocations, originating chimeric genes related to different subtypes of leukemia as reported in Mitelman et al. [14] and in Atlas of chromosome in cancer databases [15], and in the fragile site, FRA12A, which is caused by an expanded CGG repeat in the 5-prime untranslated region of the DIP2B gene (OMIM** 611379) [16]. Combining all these data we can speculate that the presence of specific genomic motif in 12q13, such as CGG repeats, could have caused the variant t(9;22) observed in our patient. To the best of our knowledge, this is the first case with this type of variant translocation in a CML patient.

We can also hypothesize that this chromosomal rearrangement was arisen by one-step mechanism with at least four simultaneous breaks and joints because (i) at diagnosis we did not detect additional clonal abnormalities and (ii) on der(22) only one breakpoint occurred, which is located within the BCR gene and that originated both the fusion gene and the t(12;22). Conversely other cases showed the coexistence of standard and complex translocation in the same patient suggesting that two or more consecutive translocations caused the formation of the complex variant translocation [4].

Prognostic data on response to Imatinib in cases with complex Philadelphia translocation are contradictory and the poor prognostic outcome in some patient of this group was explained by an increased frequency of the concomitant deletion on der(9) rather than to the type of chromosome rearrangement [5]. Our patient has been treated with Imatinib, and at 3 months of therapy she achieved the hematological and cytogenetics responses despite the presence of the deletion on der(9), while at six months of therapy she developed a clone with trisomies 8 and 9. These trisomies have apparently no prognostic significance in CML. In more detail trisomy 8 may arise after interferon and/or Imatinib treatment with unknown significance and trisomy 9 is assumed to represent a gain-of-function mechanism with respect to the JAK2 gene on 9p24 coding for the JAK2 kinase with no prognostic impact according to follow-up studies of limited sample sizes [17].

Up to now our patient showed a good response to Imatinib treatment, but further studies are needed to confirm this finding.
